# Cognitive Assessment in Chinese Patients with Aneurysmal Subarachnoid Hemorrhage

**DOI:** 10.1155/2006/728431

**Published:** 2006-07-17

**Authors:** Huilin Cheng, Jixin Shi, Mengliang Zhou

**Affiliations:** Department of NeurosurgeryJinling HospitalSchool of MedicineNanjing University; Nanjing 210002Jiangsu ProvinceChina

**Keywords:** Subarachnoid hemorrhage, neuropsychology, cognitive deficits

## Abstract

A subgroup of patients who survive aneurysmal subarachnoid hemorrhage (SAH) may have significant cognitive deficits. The aim of the current study was to determine the efficiency of cognitive tests and frequency of cognitive impairments associated with aneurysmal SAH in Chinese. A series of 116 patients with aneurysmal SAH were assessed before surgery. Only 37 patients have completed all tests. The other 79 patients had discontinued because of their clinical conditions, recurring severe headaches, refusing or misunderstanding due to low education. We found that one or more cognitive functions were impaired in 70.3% of the 37 patients, SAH patients were especially impaired in functions that are related to visual reproduction, verbal fluency, and executive functions. The results suggest that the patients have impressive cognitive deficits after aneurysmal SAH. A battery of appropriate cognitive tests should be developed for use by doctors and nurses.

## 1. Introduction

In neurosurgical clinical practice, surgical clipping and coiling of aneurysms now carries a very low risk of mortality (1%) or major physical morbidity (4%). But a subgroup of patient who survive SAH, even if having good neurological recovery, may also have significant cognitive deficits, such as disturbance of memory and attention [3,16]. Cognitive deficits in these patients can interfere with return to work and social re-integration. The Glasgow Outcome Scale (GOS), the method frequently used for evaluating the outcome of SAH patients [13], is not sensitive to the cognitive deficits of these patients [11]. Thus, evaluation of cognitive function is likely to provide a sensitive measurement to identify possible differences in outcome to due to variations in operative techniques or perioperative care [9].

It is the first time in China that a series of neuropsychological examinations was performed in patients with aneurysmal SAH before surgery, to assess the cognitive deficits of SAH patients. In this paper we report the incidence and range of cognitive deficits with aneurysmal SAH in Chinese, and evaluate the feasibility and validity of these cognitive tests.

## 2. Patients and methods

### *2.1. Sample characteristics*

The inclusion criteria of the patients were as follows: Nontraumatic SAH proven by cranial computed tomographic (CT) scanning and/or lumbar puncture; four-vessel angiographic results showing no vascular malformation other than an aneurysm; patient age between 18–70 years; and no history of serious pre-morbid medical conditions, mental disease or craniectomy. The

ISSN 0953-4180/06/$17.00 © 2006 – IOS Press and the authors. All rights reserved time interval between SAH and assessment was not more than 5 years before the examination. The severity of initial SAH bleeding visible on the computed tomography (CT) scan was not exceeding a Fisher score of III [5]. Patients were also excluded for occurrence of complications (neurological deterioration, cerebral vasospasm, hydrocephalus proven by CT scan or other medical pathologies).

During the study period, a total of 116 patients were enrolled for neuropsychological examination. Of these subjects, the distribution of aneurysms by site was as follows: 76 cases (65.5%) internal carotid artery (ICA; including aneurysms of the ICA, posterior communicating artery; and anterior choroidal artery); 29 cases (25%) anterior cerebral artery (ACA; including aneurysms of the ACA, anterior communicating artery; and pericollosal artery); and 11 cases (9.5%) middle cerebral artery. The female-to-male ratio was 47 to 69 in the patients. The age mean was $46.2 \pm 8.7$ years in the group of patients (range:18–69). The mean time from onset of SAH to being tested was 22 days (range: 1 day to 5 years). The mean education age was 7.6 years (ranged from 0 to 18 years). The neurological state at the time for tests was rated according to the grading system of Hunt and Hess (HH) [10]. Hunt-Hess (HH) grades were HH I in 23 patients, HH II in 35 patients, HH III in 32 patients, HH IV in 19 patients and HH V in 7 patients.

### 2.2. *Neuropsychological assessment*

The neuropsychological testing was performed by trained persons before the surgery, when the patients were left undisturbed by nursing care or medical interventions. For neuropsychological testing, we used a battery of standardized tests with acceptable reliability and discriminative validity and for which normative data were also available. To quantify the individual cognitive capacity for every function assessed, the individual test scores were compared with the age-adjusted test norms. The battery adopted assesses a full range of many important cognitive functions (e.g., memory, concentration, language, thinking.). The following tests were included in our neuropsychological battery: (1) the Wechsler adult intelligence scale-revised (WAIS-R) arithmetic, digits span, and digit-symbol subtests [27]; (2) the Wechsler memory scale-revised (WMS-R) picture completion, and Visual Reproduction [28]; (3) Verbal fluency test (food and commodity) [2]; (4) Trail Making Test (part A and B) [14]; (5) Mini-Mental State Examination (MMSE) [6]. All tests have been fully described in related papers.

### 2.3. *Analysis of cognitive test scores*

Cognitive deficits were identified by comparing the test scores of the patients with published age-adjusted test norms, which represent the distribution of test scores one would find in the general population [11]. A cognitive deficit was defined as a value two standard deviations or more below the mean of normal controls in Arithmetic, Digit span, Digit symbol, Picture completion, and Visual Reproduction. The verbal fluency test, Trail Making Test and the MMSE have individual cut-off values to indicate deficits [12].

## 3. Results

A total of 116 patients were included for neuropsychological examination. Only 37 patients with HH grade of I–II had completed all tests of the battery at the end of the study. Of other 79 patients, 28 patients were not testable because of their clinical conditions (including all 26 patients with HH grade of IV–V); for 33 patients, the neuropsychological examinations had to be discontinued because of recurring severe headaches triggered by mental activity, or intolerable difficulty of the tests; another six patients refused to participate in the study; 12 patients could not be included because of limited capacity to undergo the examination due to low education.

The single-case analysis of the results of individual cognitive testing in 37 patients, who have completed the full tests, showed that eleven (29.7%) had no cognitive deficits. In fourteen (37.8%) one deficit was found, and in twelve (32.4%) two or more deficits were found. In total, 70.3% of the patients were substantially impaired in some aspect of their cognitive capacity. Table 1 gives an overview of neuropsychological tests used, and the frequency of deficits revealed by these tests. In our study, SAH patients were especially impaired in functions that are related to visual reproduction, verbal fluency test and Trail Making Test.

## 4. Discussion

Most evidence for cognitive deficits has come from studies of postoperative performance of patients on neuropsychological tests [1,4,8,19,21–23,26] or questionnaires [20], or both [15,24]. The drawback of the established approach is that the cognitive deficits may have resulted from effects of brain surgery, such as

Table 1
Neuropsychological tests used and frequencies of deficits

| NeuropsychologicalTests used | value (M ± SD) | Significant deficits | |
|---|---|---|---|
| | | n | % |
| Arithmetic | 10.12 ± 4.24* | 1 | 2.7% |
| Digit span | 9.07 ± 4.15* | 2 | 5.4% |
| Digit symbol | 9.88 ± 4.34* | 1 | 2.7% |
| Picture completion | 8.69 ± 3.97* | 1 | 2.7% |
| Visual Reproduction | 3.59 ± 4.15* | 18 | 48.6% |
| Verbal fluency test (food) | 15 ± 6.59 | 10 | 27% |
| Verbal fluency test (commodity) | 14 ± 7.88 | 7 | 18.9% |
| Trail making test (A) | 103.45 ± 79.33** | 7 | 18.9% |
| Trail making test (B) | 239.36 ± 125.15** | 8 | 21.6% |
| MMSE | 25.81 ± 4.87* | 7 | 18.9% |

*: test score. **: seconds.

prolonged anaesthesia, temporary regional cessation of blood flow, brain retraction, use of antiepileptic medications, or even more general effects of bed rest and debility, rather than SAH alone [9]. It is difficult to clearly distinguish the effect of SAH or surgery, because all patients of the studies had both surgery and SAH [25]. Our study has evaluated the cognitive deficits caused by SAH itself, not the general effects of SAH and surgery. The analysis of preoperative performance on the neuropsychological tests in patients with aneurismal SAH allowed us to differentiate deficits due to SAH (and possibly associated complications such as vasospasm) versus deficits due to more general effects of neurosurgical intervention, anaesthesia, and debility. Measuring preoperative performance permitted us to determine which differences from published norms in this group were due to operative and perioperative management, and which differences are due to premorbid variability.

Like many other published papers, a control group was not used. Some studies have used normal volunteers [21,23], and orthopedic patients [26] as controls, but these groups, as McKenna et al point out, may have deleterious side-effects on cognitive function [18]. In fact, it is difficulty to find a group of subjects who had survived a medical disorder that was equally sudden and serious but did not carry the risk of brain damage. Therefore, the use of such control groups would seem to add no further information than could be gathered from normative test data [19].

The presence and patterns of cognitive impairment in patients with SAH vary between different reports. The frequency of cognitive deficits ranged from 7 to 84%, depending on the cognitive function tested. Hutter and Gilsbach found, in a sample of 31 SAH patients with a good 6-month outcome (Glasgow Outcome Scale = I), 54% with three or more cognitive deficits [11]. Bornstein et al. found 30% with relevant deficits [3]. Mavaddat et al. reported some degree of neuropsychological deficits in 47 cases who underwent open surgery for ruptured anterior communicating artery aneurysm; 40.4% were impaired on two or more tests, 43.6% on one test, and only 17% were completely unimpaired [17]. Ljunggren et al. even indicated in their sample of 40 patients with GOS = I significant deficits in 84% because they counted all patients with at least one deficit [16]. In nearly all studies, SAH patients were especially impaired in functions that are related to cognitive speed, concentration capacity, and memory function without a reduction of general intelligence or global intellectual functions [11]. Our result indicated that deficits of attention, executive function, and memory are prominent in SAH patients.

We found that these cognitive tests, which have been used in many investigations, are not suited for the majority of patients with SAH. Although the tests have been modified in our study for use in Chinese patients, many patients dropped out. About 68% (79 of 116) patients could not complete all the tests of the battery. Of these, some patients had to be excluded by their unstable conditions or discontinued because of recurring severe headaches, time-lasting and difficulty of performances. Some tests, such as vocabulary, cannot be retested in short period because these tests can be remembered. The verbal tests were difficult in some patients who were uneducated. Our results were similar to those of Hutter, et al., who reported that 51% (53 of 104) patients dropped out of neuropsychological tests [12]. Many reports of cognitive deficits following SAH were administrated in a selection of patients who were in relatively good condition [3,7,11,16,17]. Therefore, and the conclusions may not apply to all patients after aneurysmal SAH [12].

Therefore, we recommend that reasonable neurocognitive screening in clinical practice should have the following characteristics: (1) multiple cognitive domains evaluated; (2) reliable and valid for identifying cognitive deficits; (3) potential for grading the severity of cognitive deficits; (4) rapidly testable at bedside within a few minutes; and (5) requiring minimal training.

In summary, our results provide preliminary evidence that SAH itself may result in cognitive deficits in Chinese. Additional research is required to develop a battery of cognitive tests appropriate for use in clinic by doctors and nurses.

## Acknowledgments

The authors wish to thank the participants and the staff at Department of Neurosurgery, Jinlin Hospital.
